# High *IL2RA* mRNA expression is an independent adverse prognostic biomarker in core binding factor and intermediate-risk acute myeloid leukemia

**DOI:** 10.1186/s12967-019-1926-z

**Published:** 2019-06-06

**Authors:** Wen Du, Jing He, Wei Zhou, Simin Shu, Juan Li, Wei Liu, Yun Deng, Cong Lu, Shengyan Lin, Yaokun Ma, Yanli He, Jine Zheng, Jiang Zhu, Lijuan Bai, Xiaoqing Li, Junxia Yao, Dan Hu, Shengqing Gu, Huiyu Li, Anyuan Guo, Shiang Huang, Xiaolan Feng, Dong Hu

**Affiliations:** 1 Center for Stem Cell Research and Application, Union Hospital, Tongji Medical College, Huazhong University of Science and Technology, 1277 Jiefang Avenue, Wuhan, 430022 Hubei China; 2 Institute of Hematology, Union Hospital, Tongji Medical College, Huazhong University of Science and Technology, Wuhan, 430022 China; 3 Biological Targeted Therapy Key Laboratory in Hubei, Wuhan, 430022 China; 4 Wuhan Kindstar Diagnostics, Wuhan, 430075 China; 5 Department of Bioinformatics and Systems Biology, Key Laboratory of Molecular Biophysics of the Ministry of Education, College of Life Science and Technology, Huazhong University of Science and Technology, Wuhan, 430074 China; 6 Department of Geriatrics, Union Hospital, Tongji Medical College, Huazhong University of Science and Technology, Wuhan, 430022 China; 7 Department of Cardiology and Cardiovascular Research Institute, Renmin Hospital of Wuhan University, Wuhan, 430060 China; 8 Department of Medical Oncology, Dana-Farber Cancer Institute, Boston, MA USA; 9 BC Cancer Victoria, Victoria, BC V8R 6V5 Canada

**Keywords:** Acute myeloid leukemia, Prognosis, *IL2RA*, mRNA expression, Core binding factor AML, Intermediate-risk AML, NanoString

## Abstract

**Background:**

Elevated protein expressions of CD markers such as IL2RA/CD25, CXCR4/CD184, CD34 and CD56 are associated with adverse prognosis in acute myeloid leukemia (AML). However, the prognostic value of mRNA expressions of these CD markers in AML remains unclear. Through our pilot evaluation, *IL2RA* mRNA expression appeared to be the best candidate as a prognostic biomarker. Therefore, the aim of this study is to characterize the prognostic value of *IL2RA* mRNA expression and evaluate its potential to refine prognostification in AML.

**Methods:**

In a cohort of 239 newly diagnosed AML patients, *IL2RA* mRNA expression were measured by TaqMan realtime quantitative PCR. Morphological, cytogenetics and mutational analyses were also performed. In an intermediate-risk AML cohort with 66 patients, the mRNA expression of prognostic biomarkers (*BAALC*, *CDKN1B*, *ERG*, *MECOM/EVI1*, *FLT3*, *ID1*, *IL2RA, MN1* and *WT1*) were quantified by NanoString technology. A TCGA cohort was analyzed to validate the prognostic value of *IL2RA*. For statistical analysis, Mann–Whitney U test, Fisher exact test, logistic regression, Kaplan–Meier and Cox regression analyses were used.

**Results:**

In AML cohort of 239 patients, high *IL2RA* mRNA expression independently predicted shorter relapse free survival (RFS, p < 0.001) and overall survival (OS, p < 0.001) irrespective of age, cytogenetics, *FLT3*-*ITD* or *c*-*KIT D816V* mutational status. In core binding factor (CBF) AML, high *IL2RA* mRNA expression correlated with *FLT3*-*ITD* status (p = 0.023). Multivariable analyses revealed that high *IL2RA* expression (p = 0.002), along with *c*-*KIT D816V* status (p = 0.013) significantly predicted shorter RFS, whereas only high *IL2RA* mRNA expression (p = 0.014) significantly predicted shorter OS in CBF AML. In intermediate-risk AML in which multiple gene expression markers were tested by NanoString, *IL2RA* significantly correlated with *ID1* (p = 0.006), *FLT3* (p = 0.007), *CDKN1B* (p = 0.033) and *ERG* (p = 0.030) expressions. *IL2RA* (p < 0.001) and *FLT3* (p = 0.008) expressions remained significant in predicting shorter RFS, whereas *ERG* (p = 0.008) and *IL2RA* (p = 0.044) remained significant in predicting shorter OS. Similar analyses in TCGA intermediate-risk AML showed the independent prognostic role of *IL2RA* in predicting event free survival (p < 0.001) and OS (p < 0.001).

**Conclusions:**

High *IL2RA* mRNA expression is an independent and adverse prognostic factor in AML and specifically stratifies patients to worse prognosis in both CBF and intermediate-risk AML.

**Electronic supplementary material:**

The online version of this article (10.1186/s12967-019-1926-z) contains supplementary material, which is available to authorized users.

## Background

Acute myeloid leukemia (AML) is a well-known heterogeneous hematological malignancy with a broad range of prognosis, which is greatly impacted by clinical factors, cytogenetics and molecular characteristics [1]. In the current risk stratification system, recurrent genetic abnormalities stratify AML into three risk status categories including favourable-risk, intermediate-risk and poor-risk [1]. This risk stratification system, together with clinical characteristics of AML patients such as age and medical comorbid, dictates the prognosis of each individual patient, as well as guide physicians to decide appropriate treatment regimens [1]. For instance, in younger adult patients high-dose cytarabine-based therapy has been recommended as the conventional consolidation regimen for AML of favourable-risk, whereas allogeneic hematopoietic stem cell transplantation (HCT) for AML of poor-risk [1]. Nevertheless, either regimen can be considered for AML of intermediate-risk [1]. While patients have benefited greatly from current risk stratification strategies of AML, the prognosis of AML in each risk category is still quite variable. Further improvement of prognostic tools is needed to better stratify these patients and guide treatments accordingly. In addition to the well-established recurrent cytogenetic aberrations and molecular mutations, genes with aberrant expression at protein or mRNA level have also been shown to have significant prognostic values in AML over the last decade [2]. These gene expression biomarkers not only help shed light on mechanisms of development and progression of this largely heterogeneous malignancy, but more importantly help clinicians to refine prognostic tools to improve patient care in clinical practice.

Elevated protein expressions of various cluster of differentiation (CD) marker genes such as *interleukin 2 receptor subunit alpha* (*IL2RA/CD25*) [3–7], *C*-*X*-*C chemokine receptor type 4* (*CXCR4/CD184*) [8–10], *CD34* [11, 12] and *CD56* [13, 14] have been shown to predict poor clinical outcome in AML. Most studies used flow cytometry (FCM) technology to quantify protein expression of these CD markers. Meanwhile, many studies based on mRNA quantification platforms identified mRNA expression biomarkers in AML, such as *BAALC*, *ERG*, *MECOM/EVI1* and *WT1*, which could offer additional prognostic values to improve current stratification system [15–21]. RNA quantitative methodology, in comparison to FCM method, have several unique advantages. Firstly, stored RNA or bone marrow (BM) samples could be used to accurately quantify RNA gene expression when fresh samples are not available. This enhanced tissue flexibility would potentially improve patient care in practice. In addition, this approach makes retrospective analysis of samples feasible, which also enhance clinical care and facilitate research. Secondly, only limited tissue is needed for RNA quantitation that could significantly improves tissue efficiency. Lastly, many RNA quantitative platforms are designed to perform multiplex gene testing to improve consistency and efficiency.

By far, the prognostic values of mRNA expression of most CD markers in AML remain elusive. To our knowledge, the only prognostic CD marker, of which the mRNA expression prognostic value has been indicated, is CD34 [22, 23]. However, reports on prognostic values of *CD34* mRNA and protein expressions were not consistent [24–26]. In general, gene expression levels at mRNA level do not necessarily correlate well with those at protein level [27, 28] which are subject to multiple layers of regulation [27–29]. In addition, the protein levels of known CD biomarkers in AML are generally quantitated by FCM assay on blast cells, whereas mRNA expression levels of CD markers are quantitated in bulk tissue using different platforms. Therefore, it is important to investigate and validate the prognostic value of these CD biomarkers at mRNA level independently.

In our study, we initially sought to investigate the prognostic value of mRNA expressions of various CD biomarkers in AML and study if they can add independent prognostic value to the established prognostic factors. We conducted a pilot study to evaluate correlations of mRNA/protein expressions of four prognostic CD marker genes (*IL2RA*, *CXCR4*, *CD34* and *CD56*), analyzed the prognostic values of their mRNA expressions in the TCGA-LAML cohort and determined *IL2RA* as best candidate gene for further study in larger cohort. Subsequently, in our clinical cohorts, we aimed to systemically evaluate the prognostic value of *IL2RA* mRNA expression in AML in the context of clinical and laboratory factors with prognostic relevance. We further characterized its prognostic role in core binding factor (CBF) AML in the context of established prognostic factors, as well as in intermediate-risk AML in the context of other mRNA expression prognostic factors (*ERG*, *ID1*, *WT1*, *FLT3*, *WT1*, *BAALC*, *CDKN1B*, *MECOM/EVI1*, *MN1*). Our study not only consistently reveals independent prognostic value of mRNA expression of *IL2RA* in AML, particularly in CBF and intermediate-risk AML, but also serves as a proof-of-concept study for future research endeavors to investigate prognostic roles of mRNA expression of other CD biomarkers.

## Methods

### Patients and treatments

We analyzed mRNA expression of *IL2RA* using BM samples from 239 adult patients (age range: 15–65) diagnosed with AML between 2012 and 2016 at Institute of Hematology, Wuhan Union Hospital. The patients received intensive induction chemotherapy and consolidation chemotherapy or HCT [1]. Cases of acute promyelocytic leukemia (APL) were not included in this cohort. We also analyzed expression of *IL2RA* together with eight other known prognostic genes simultaneously in a multigene panel testing platform by NanoString using BM samples from 66 adult patients diagnosed with intermediate-risk AML. The diagnosis of AML was made according to World Health Organization classification [30] and French–American–British (Fab) classification. Cytogenetic risk stratification was defined according to the AML NCCN guideline version 3. 2017. This study was approved by the ethics committee of Tongji Medical College, Huazhong University of Science and Technology and was carried out in accordance with the Helsinki Declaration.

### Cytogenetic analysis

Conventional cytogenetic analysis was performed on G-banded preparations from 48-h bone marrow cell cultures. The chromosomal aberrations were described according to the International System for Cytogenetic Nomenclature (ISCN) 2009 [31].

### Flow cytometry

Flow cytometry analysis on fresh bone marrow samples were carried out at the time of diagnosis. In addition to monoclonal antibodies (Abs) and isotype control IgGs used for diagnostic immunophenotyping [32], antibodies for IL2RA/CD25 (347643, BD Bioscience, CA) and CXCR4/CD184 (555976, BD Bioscience Pharmingen, CA) were used. Flow cytometry analyses were performed as previously described [32].

### DNA extraction and mutational analyses

Genomic DNA was extracted from diagnostic marrow specimens BM samples using standard method and *FMS*-*like tyrosine kinase 3*-*internal tandem duplication (FLT3*-*ITD)* [33], *nucleophosmin 1 (NPM1)* [34], *CEBPA* [35] and *c*-*KIT D816V* [36] mutation status were evaluated as previously described. Direct sequencing of DNA was performed using Thermo Sequence Dye Terminator sequencing reaction and ABI Prism 3730 sequencing analyzer (Thermo Fisher Scientific, Carlsbad, CA).

### RNA extraction and TaqMan RQ-PCR

BM samples RNA extraction were performed as previously described [37]. Reverse transcription and cDNA synthesis were prepared from ~ 1.0 μg of total RNA using the PrimeScript RT Master Mix (Takara Bio Inc, Japan) according to the manufacturer’s instruction and 1/8 of the cDNA was used as a template for each PCR reaction. PCR reactions and fluorescence quantitative measurements were conducted on the Applied Biosystems Prism 7500 instrument (PE Applied Biosystems, USA). TaqMan Fast Advanced Master Mix (ThermoFisher Scientific, USA) were used to perform the TaqMan assay according to the manufacturer’s instruction. The *IL2RA* gene expression was measured by TaqMan assay Hs00907779_m1 and the *CXCR4* gene expression was measured by TaqMan assay as previously described [38]. *ABL1* gene was selected as the reference gene to compensate for the variations in mRNA and cDNA, and the *ABL1* TaqMan assay was described in previous study [39]. The comparative cycle threshold (Ct) method was used to calculate the relative expression of target genes [40]. The threshold cycles for *IL2RA*, *CXCR4* and *ABL1* were determined by replicates and the mean C_T_ was used for calculation. The cycle number difference was calculated (ΔCt = Ct target gene − Ct*ABL1*) and the relative expression values of target gene were expressed as 2^−ΔCT^. The PCR efficiencies of *IL2RA* and *ABL1* assays have been evaluated by standard curves. The standard curves for *ABL1* assays were generated by tenfold dilution series of four different plasmid concentrations (copy numbers 10^3^; 10^4^; 10^5^; 10^6^) and the standard curves for *IL2RA* assays were generated by tenfold dilution series of positive RNA samples (1; 1:10; 1:10^2^; 1:10^3^). The PCR efficiencies calculated for either *ABL1* or *IL2RA* assays varied between 95.0 and 105.0%. Negative controls and interassay positive controls have been included in the PCR assays. The data were analyzed using the Applied Biosystems 7500 software v2.0.5.

### NanoString nCounter assay

The probes sequences for the prognostic transcripts including the reference gene *ABL1* were designed according to principles described before [41]. Six positive and eight negative spike-in control probes were included in the probes codeset and were used for normalization of raw data. The counts of the reference gene *ABL1* were used to further normalize the gene counts. For each NanoString assay, about 250 ng sample RNA was used for each reaction. The experimental procedures were carried out on the NanoString Preparation Station and Digital Analyzer according to manufacturer’s instructions [37, 41]. The nSolver 3.0 software were used for analyses.

### TCGA dataset

Level 3 RNA-seq gene expression data (fragments per kilobase per million mapped fragments/FPKM values) of 151 AML cases were retrieved from the TCGA data portal and clinical data from the cohort were retrieved from Additional file 1: Table S1 of the publication of the TCGA-LAML study [42]. Of the 151 cases, there are 136 cases of AML cases excluding APL and 80 cases of intermediate-risk AML.

### Statistical analysis

Mann–Whitney U test or Kruskal–Wallis test was used for comparison of continuous variable between different subsets of patients. Fisher exact or Chi-square tests were used for comparison of categorical variables between different subsets of patients. The bivariate Spearman correlation test was used to measure associations among continuous variables. Normalized NanoString counts were log2 transformed before the correlation analysis. Complete remission (CR), RFS and OS were defined as previous described [43]. Univariable logistic regression models were performed to evaluate the achievement of CR. Univariable survival analyses using Kaplan–Meier method were performed to evaluate association of RFS or OS for dichotomous variables, the differences were calculated by log-rank test. Univariable survival analyses for continuous variables was performed using Cox regression method. Multivariable logistic regression models were generated for CR and multivariable Cox regression models for RFS and OS. The statistical analyses were performed using IBM SPSS 19.0.0.

## Results

### The pilot study revealed the mRNA expressions of *IL2RA* is the most suitable prognostic CD marker gene

**Fig. 1:**
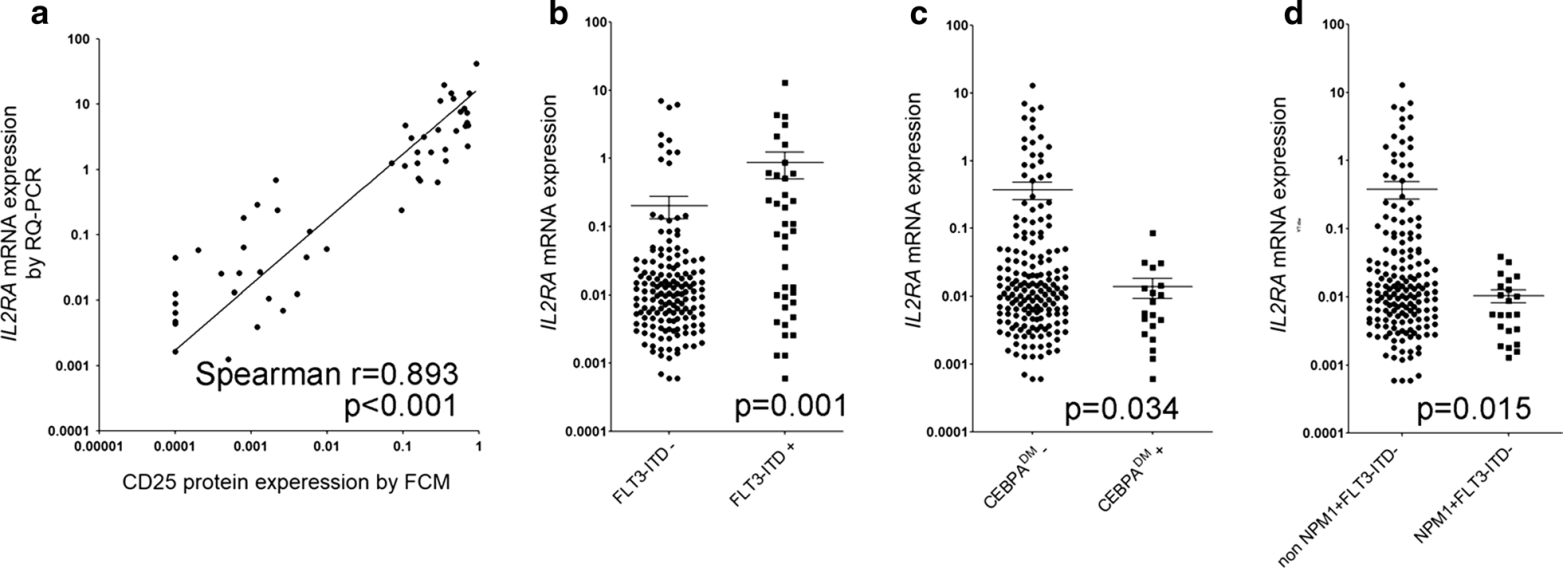
Correlation of mRNA/protein expressions of *IL2RA* and *IL2RA* mRNA expression levels according to mutational status. **a** Correlation of *IL2RA* mRNA expression with its protein expression is significant, p < 0.001 and correlation coefficient Spearman r = 0.893. **b** There is much higher level of *IL2RA* and in *FLT3*-*ITD*+ group than in *FLT3*-*ITD*− group, p = 0.001. **c** There is much lower level of *IL2RA* mRNA expression and in *CEBPA*^*DM*^ group than in non-*CEBPA*^*DM*^ group, p = 0.034. **d** There is much lower level of *IL2RA* mRNA expression in *NPM1*+*FLT3*-*ITD*− group than in non*NPM1*+*FLT3*-*ITD*− group, p = 0.015

In order to determine the suitable prognostic biomarker candidate genes, we performed initial evaluation of both mRNA as well as protein levels on four CD marker genes (*IL2RA*, *CXCR4*, *CD56* and *CD34*). The *IL2RA* mRNA and its protein CD25 levels were assayed in 54 primary AML BM samples by TaqMan RQ-PCR and FCM respectively, in which the mRNA level was shown as 2^−∆Ct^ of *IL2RA* gene relative to *ABL1* gene and the expression level of CD25 protein was illustrated as percentage in leukemia blast cells. A significant association was found between mRNA expression of *IL2RA* gene and protein expression of CD25 (p < 0.001 and correlation coefficient Spearman r = 0.893, Fig. 1a and Additional file 1: Table S1). Although the percentage of leukemia blasts varies from 20 to 99% in the diagnostic bone marrow specimens of this study group, *IL2RA* expression level was independent of the blast percentage (p = 0.626, Spearman r = − 0.0679, Additional file 1: Fig. S1). The *CXCR4* mRNA and its protein CD184 expressions were examined in 41 adult AML patients by TaqMan RQ-PCR and FCM, whereas no significant association was found (p = 0.863 and Spearman r = 0.0278, Additional file 1: Fig. S2 and Table S1). Correlations between mRNA and protein expressions of *CD34* and *CD56* genes have been studied and reported previously in two independent studies using leukemia samples [44, 45] (Additional file 1: Table S1). Significant correlation of *CD34* mRNA/protein expressions has been shown in both studies, whereas inconsistent results were indicated for *CD56* (p < 0.01 and p = 0.180 respectively, Additional file 1: Table S1).

Next, we performed univariable Cox regression analysis to evaluate prognostic role of mRNA expressions of the four CD markers genes as continuous variables on OS in an existing TCGA-LAML cohort (2013). Among these CD markers, only the mRNA expression of *IL2RA/CD25* seemed to significantly predict OS in AML (p < 0.001, Additional file 1: Table S1). Therefore, we selected mRNA expression of *IL2RA* as the best suitable candidate prognostic biomarker for further validation analysis in a larger AML cohort.

### Patients characteristics of AML cohort and association with *IL2RA* mRNA expression

In a cohort of 239 newly diagnosed AML adult patients, we quantified the *IL2RA* mRNA expression using BM RNA samples by TaqMan RQ-PCR. The characteristics of patients including clinical, cytogenetics and molecular features in this cohort were described and their correlation with *IL2RA* mRNA expression level were analyzed and summarized in Table 1. Significant differences of *IL2RA* mRNA expression were observed in AML with certain molecular abnormalities (Table 1). In particular, a positive correlation of *IL2RA* mRNA expression level with *FLT3*-*internal tandem duplication* (*FLT3*-*ITD*) mutation (p = 0.001, Table 1 and Fig. 1b) and a negative correlation with *CEBPA double mutations* (*CEBPA*^*DM*^) (p = 0.034, Table 1 and Fig. 1c) or *NPM1* mutation in the absence of *FLT3*-*ITD*(*NPM1*+*FLT3*-*ITD*−, p = 0.015, Table 1 and Fig. 1d) were found.

**Table 1:** Correlation of *IL2RA* mRNA expression with clinical and laboratory parameters in AML cohort

	N	Percentage (%)	*IL2RA* mRNA expression range	Median	*IL2RA* mRNA expression Mean ± SE	p-value
Total	239					
Age group						0.312
15–29	65	27.2	0.0014–3.780	0.0128	0.211 ± 0.603	
30–49	113	47.3	0.0006–7.056	0.0097	0.304 ± 1.092	
50–65	61	25.5	0.0013–12.870	0.0130	0.598 ± 2.184	
Gender						0.973
Male	127	53.1	0.0006–12.870	0.0105	0.395 ± 1.573	
Female	112	46.9	0.0006–6.140	0.0122	0.306 ± 1.108	
Blast percentage (%)						0.941
≥ 50	149	62.3	0.0006–8.540	0.0113	0.349 ± 1.247	
20–49	90	37.7	0.0006–12.870	0.0114	0.361 ± 1.566	
WBC count						0.684
≥ 30 billion/L; high	58	34.7	0.0007–5.890	0.0136	0.341 ± 1.060	
< 30 billion/L; low	109	65.3	0.0006–12.870	0.0106	0.430 ± 1.649	
AML type						0.605
De novo AML	231	96.7	0.0006–12.870	0.0113	0.353 ± 1.392	
Secondary AML	8	3.3	0.0033–1.560	0.0258	0.365 ± 0.642	
Fab subtypes						0.279
Fab M0	6	2.6	0.0026–0.0468	0.0123	0.0172 ± 0.0161	
Fab M1	32	13.6	0.0013–6.140	0.0136	0.402 ± 1.474	
Fab M2	118	50.2	0.0006–4.120	0.0096	0.146 ± 0.576	
Fab M4	43	18.3	0.0007–12.870	0.0175	0.605 ± 2.097	
Fab M5	26	11.1	0.0006–8.540	0.0060	0.689 ± 1.981	
Fab M6	10	4.3	0.0020–7.056	0.0521	0.749 ± 2.216	
ND	4					
Cytogenetics groups						0.141
Favorable-risk (CBF)	54	22.6	0.0006–3.780	0.0137	0.138 ± 0.543	
Intermediate-risk	148	61.9	0.0006–12.870	0.0096	0.417 ± 1.547	
Poor-risk	37	15.5	0.0026–7.056	0.0170	0.416 ± 1.473	
*Flt3*-*ITD*						0.001
*Flt3*-*ITD*+	38	19.8	0.0006–12.870	0.0820	0.871 ± 2.275	
*Flt3*-*ITD−*	154	80.2	0.0006–7.056	0.0105	0.205 ± 0.918	
*NPM1*						0.818
*NPM1*+	36	18.8	0.0013–12.870	0.0117	0.452 ± 2.137	
*NPM1−*	156	81.2	0.0006–7.056	0.0114	0.310 ± 1.058	
*NPM1*+*FLT3*-*ITD*−						0.015
*NPM1*+*FLT3*-*ITD*−	22	11.4	0.0013–0.0386	0.0056	0.0104 ± 0.0102	
*NonNPM1*+*FLT3*-*ITD*−	170	88.6	0.0006–12.870	0.0128	0.377 ± 1.396	
*CEBPA*						0.034
*CEBPA*^*DM*^+	19	9.9	0.0006–0.0848	0.0056	0.0138 ± 0.0197	
*CEBPA*^*DM*^−	173	90.1	0.0006–12.870	0.0126	0.372 ± 1.389	

N: number; ND: not determined; *CEBPA*^*DM*^: *CEBPA double mutations*

In order to determine high versue low *IL2RA* mRNA expression levels, we used a minimal *p*-value approach and comprehensively analyzed on correlations of *IL2RA* expression with clinical outcome endpoints (CR, RFS and OS). Based on the results, we set *IL2RA* mRNA expression cutoff value at 80 percentiles (Additional file 1: Table S2). Correlation analysis between patients’ characteristics with *IL2RA* mRNA expression level as dichotomous variable were then performed. There was significantly higher frequencies of high *IL2RA* mRNA level cases in patients with *FLT3*-*ITD* mutation (p < 0.001, Table 3) or with *t(16;21);FUS*-*ERG* (p = 0.040, Table 2) abnormality and there was significantly lower frequencies of high *IL2RA* mRNA level cases in patients with *NPM1*+*FLT3*-*ITD*− mutational status (p = 0.005, Table 2). There was no significant association between *IL2RA* mRNA expression level and other cytogenetic or genetic abnormalities (Table 2) or other general features of patients’ characteristics (Additional file 1: Table S3).

**Table 2:** Correlation of *IL2RA* mRNA expressions with cytogenetical and genetic abnormalities in AML

Cytogenetical and genetic abnormalities	N/Total	Low *IL2RA* mRNA expression	High *IL2RA* mRNA expression	p-value
CBF	54/239	45/191	9/48	0.565
Intermediate-risk	148/239	118/191	30/48	1.000
Poor-risk	39/239	29/191	9/48	0.505
t(8;21);*RUNX1*-*RUNX1T1*	37/239	29/191	8/48	0.824
inv(16) or t(16;16);*CBFB*-*MYH11*	17/239	16/191	1/48	0.207
t(9;11);*MLLT3*-*KMT2A*	4/239	4/191	0/48	0.586
t(6;9);*DEK*-*NUP214*	3/239	2/191	1/48	0.491
inv(3) or t(3;3);G*ATA2, MECOM*	6/239	4/191	2/48	0.347
11q23; non t(9;11)	3/239	3/191	0/48	0.700
t(16;21);*FUS*-*ERG*	2/239	0/191	2/48	0.040
−7/7q−	11/239	7/191	4/48	0.238
−5/5q−	5/239	4/191	1/48	1.000
Complex	17/239	12/191	5/48	0.346
Normal karyotype	113/239	92/191	21/48	0.630
Trisomy	17/239	13/191	4/48	0.754
*FLT3*-*ITD*+	38/192	17/151	21/41	< 0.001
*NPM1*+	36/192	28/151	8/41	1.000
*NPM1*+*FLT3*-*ITD*−	22/192	22/151	0/41	0.005
*CEBPA*^*DM*^+	19/192	18/151	1/41	0.082
*c*-*KIT D816V*+	7/192	6/151	1/41	1.000

N: number

### Correlation of high *IL2RA* mRNA level with poor clinical outcomes in AML cohort

**Fig. 2:**
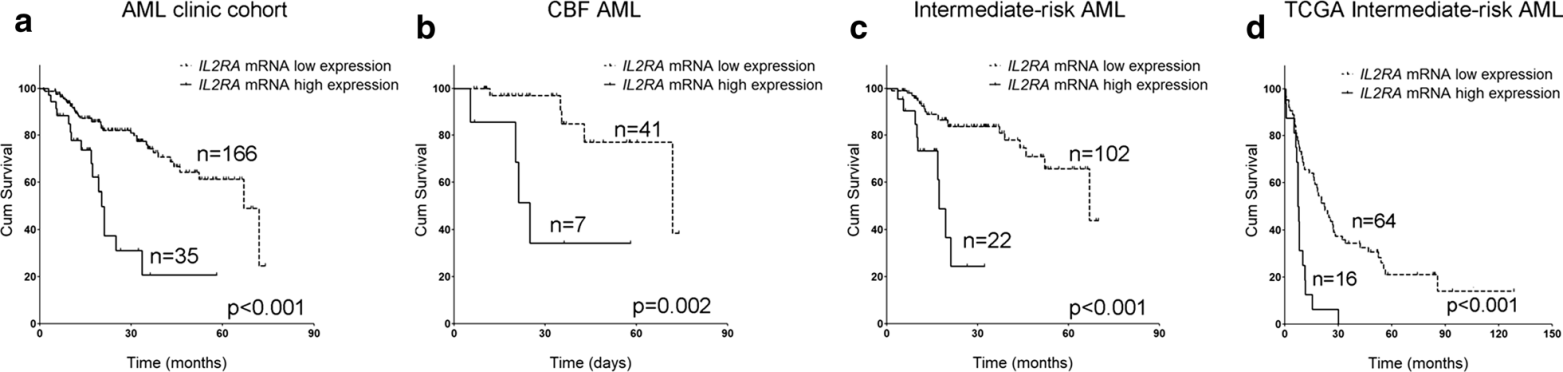
Survival analysis of OS by *IL2RA* mRNA expression levels in AML cohorts. **a** In our AML clinical cohort, the OS of patients with higher *IL2RA* expression are significantly shorter than those with lower expression levels (p < 0.001), with the median OS 20.3 months vs. 66.9 months. **b** In CBF AML, the OS of patients with higher *IL2RA* expression are significantly shorter than those with lower expression levels (p = 0.002), with the median OS 25.0 months vs. 71.8 months. **c** In intermediate-risk AML, the OS of patients with higher *IL2RA* expression are significantly shorter than those with lower expression levels (p < 0.001), with the median OS 17.3 months vs. 66.9 months. **d** In 80 TCGA intermediate-risk AML cases, higher *IL2RA* expression showed significantly shorter OS (p < 0.001), with the median OS 7.5 months vs. 22.3 months

We further sought to examine if mRNA expression of *IL2RA*, in the context of other known important clinical or laboratory markers, correlates with clinical outcomes including CR status after two cycles of induction chemotherapy, RFS and OS in the cohort of 239 AML patients. Univariable analyses showed that high *IL2RA* mRNA level significantly correlated with CR status (p = 0.005) shorter RFS (p < 0.001) and shorter OS (p < 0.001, Table 3 and Fig. 2a). Other parameters, such as age, AML type (secondary vs. de novo), cytogenetics risk group, *FLT3*-*ITD* or *c*-*KIT D816V* mutation status also appeared to be significant predictors of clinical outcomes (Table 3). The prognostic value of *IL2RA* mRNA expression level was further confirmed by multivariable analyses, which showed that high *IL2RA* expression (p = 0.008, HR = 2.617) and cytogenetics risk status (p = 0.010, HR = 2.024) remained significant in predicting CR status, high *IL2RA* expression (p < 0.001, HR = 4.008), cytogenetics risk status (p = 0.045, HR = 1.400) and *c*-*KIT D816V* (p = 0.015, HR = 3.145) remained significant in predicting shorter RFS and high *IL2RA* expression (p < 0.001, HR = 3.448), cytogenetics risk group (p < 0.001, HR = 2.417) and age (p = 0.040, HR = 1.485) remained significant in predicting shorter OS (Table 3).

**Table 3:** Uni and multivariable analysis on CR status, RFS and OS in AML cohort

Variable	Univariable			Multivariable					
	CR	RFS	OS	CR		RFS		OS	
	p-value	p-value	p-value	p-value	HR (95% CI)	p-value	HR (95% CI)	p-value	HR (95% CI)
Age group	0.038	0.306	0.002					0.040	1.485 (1.017–2.168)
High WBC counts	0.880	0.603	0.929						
High blast percentage	0.443	0.173	0.964						
Secondary AML	0.023	0.243	0.010						
Cytogenetics risk groups	0.006	0.001	< 0.001	0.010	2.024 (1.188–3.449)	0.045	1.400 (1.007–1.947)	< 0.001	2.417 (1.480–3.948)
High *IL2RA* mRNA level	0.005	< 0.001	< 0.001	0.008	2.17 (1.281–5.346)	< 0.001	4.008 (2.437–6.592)	< 0.001	3.448 (1.839–6.462)
*FLT3*-*ITD*+	0.183	0.019	0.018						
*NPM1*+	0.963	0.955	0.507						
*NPM1*+*FLT3*-*ITD*−	0.759	0.269	0.291						
*c*-*KIT D816V*+	0.845	0.022	0.668			0.015	3.145 (1.251–7.0901		
*CEBPA*^*DM*^+	0.998	0.813	0.061

### High *IL2RA* mRNA expression level predicted adverse clinical outcome in core binding factor (CBF) AML and intermediate-risk AML

Within our cohort, AML of favourable-risk group only contain CBF AML with t(8;21);RUNX1-RUNX1T1 or inv(16);CBFB-MYH11 cytogenetics, since APL were not included. In the AML cohort, there were 9 out of 54 (16.7%) cases with high *IL2RA* expression in CBF AML subgroup, 30 out of 145 (20.3%) cases with high *IL2RA* expression in intermediate-risk AML and 9 out of 37 (24.3%) cases in poor-risk AML subgroup. Although high *IL2RA* expression cases appeared to be more frequent in AML of poor risk than in AML of favourable- or intermediate-risk, the difference was not significant (p = 0.667, Additional file 1: Table S3). We further characterized the prognostic value of *IL2RA* mRNA expression in CBF AML, intermediate-risk AML and poor-risk AML.

Within the CBF AML subgroup, *IL2RA* mRNA expression status was significantly associated with age (p = 0.026) and *FLT3*-*ITD* (p = 0.023) status (Table 4), but not with *c*-*KIT D816V* mutation (p = 1.000, Table 4). In CBF AML, univariable analyses by Kaplan–Meier method showed that high *IL2RA* mRNA level correlated with shorter RFS (p < 0.001) and shorter OS (p = 0.001, Table 5 and Fig. 2b). Further multivariable analyses confirmed high *IL2RA* mRNA level (p = 0.002, HR = 5.872), along with *c*-*KIT D816V* (p = 0.013, HR = 4.309) remained as significant in predicting shorter RFS and high *IL2RA* mRNA level (p = 0.014, HR = 5.718) alone remained significant in predicting shorter OS in this particular subtype of AML (Table 5).

**Table 4:** Association of *IL2RA* mRNA expression with prognosis relevant variables in CBF, intermediate-risk and poor-risk AML

	CBF AML			Intermediate-risk AML			Poor-risk AML		
	High *IL2RA* mRNA	Low *IL2RA* mRNA	p-value	High *IL2RA* mRNA	Low *IL2RA* mRNA	p-value	High *IL2RA* mRNA	Low *IL2RA* mRNA	p-value
Age group			0.026			0.813			0.348
15–29	5 (9)	16 (45)		8 (30)	26 (118)		1 (9)	9 (28)	
30–49	1 (9)	25 (45)		15 (30)	59 (118)		3 (9)	10 (28)	
50–65	3 (9)	4 (45)		7 (30)	33 (118)		5 (9)	9 (28)	
High WBC counts	2 (8)	12 (30)	0.686	9 (18)	29 (78)	0.423	1 (9)	5 (23)	0.648
High blast percentage	5 (9)	32 (45)	0.439	18 (30)	72 (118)	1.000	3 (9)	19 (28)	0.118
Secondary AML	0 (9)	0 (45)	NA	1/30	2/118	0.496	2/9	3 (28)	0.577
*FLT3*-*ITD*+	2 (7)	0 (36)	0.023	17 (27)	14 (90)	< 0.001	2 (7)	3 (25)	0.296
*NPM1*+	0 (7)	0 (36)	NA	8 (27)	28 (90)	1.000	0 (7)	0 (25)	NA
*NPM1*+*FLT3*-*ITD*−	0 (7)	0 (36)	NA	0 (27)	22 (90)	0.002	0 (7)	0 (25)	NA
*CEBPA*^*DM*^+	0 (7)	0 (36)	NA	1 (27)	18 (90)	0.070	0 (7)	0 (25)	NA
*c*-*Kit D816V*+	1 (7)	4 (36)	1.000	0 (27)	1 (90)	1.000	0 (7)	1 (25)	1.000

The number in the parentheses is the number of patients that are of high or low *IL2RA* expression tested in that particular parameter

*NA* not applicable

*The p value for RFS and OS is calculated by Kaplan–Meier log-rank test. The p value for the other parameters are calculated by Fisher’s exact test or Chi-Square test

**Table 5:** Uni and multivariable analyses on RFS and OS in CBF AML

Variable	Univariable		Multivariable			
	RFS	OS	RFS		OS	
	p value	p value	p value	HR (95% CI)	p value	HR (95% CI)
Age group	0.549	0.053				
High WBC counts	0.343	0.197				
High blast percentage	0.056	0.344				
High *IL2RA* mRNA level	< 0.001	0.002	0.002	5.872 (1.902–18.129)	0.014	5.718 (1.423–22.984)
*FLT3*-*ITD*+	0.526	0.017				
*c*-*Kit D816V*+	0.006	0.398	0.013	4.309 (1.365–13.607)		

Within the intermediate-risk AML subgroup, high *IL2RA* mRNA expression level was significantly associated with *FLT3*-*ITD* (p < 0.001, Table 5) and *NPM1*+*FLT3*-*ITD*− (p = 0.002, Table 5) mutational status. High *IL2RA* mRNA expression significantly correlated with lower CR rate (p = 0.018, Table 5), shorter RFS (p < 0.001) and shorter OS (p < 0.001) by univariable analysis (Table 6 and Fig. 2c). As well, high *IL2RA* mRNA level remain as significant predictor for shorter RFS (p < 0.001, HR = 6.637) and shorter OS (p < 0.001, HR = 5.211, Table 6 and Fig. 2c) by multivariable analyses within this subgroup of AML.

**Table 6:** Uni and multivariable analyses on RFS and OS in intermediate-risk AML

Variable	Univariable		Multivariable			
	RFS	OS	RFS		OS	
	p value	p value	p value	HR (95% CI)	p value	HR (95% CI)
Age group	0.633	0.223				
High WBC counts	0.512	0.300				
High blast percentage	0.759	0.779				
Secondary AML	0.705	0.168				
High *IL2RA* mRNA level	< 0.001	< 0.001	< 0.001	6.637 (3.424–12.865)	< 0.001	5.211 (2.129–12.752)
*FLT3*-*ITD*+	0.006	0.006				
*CEBPA*^*DM*^+	0.930	0.071				
*NPM1*+	0.685	0.898				
*NPM1*+*FLT3*-*ITD*−	0.440	0.478				

Within the poor-risk AML subgroup, however, high *IL2RA* mRNA expression was not associated with relevant prognostic clinical or laboratory parameters (Table 4) nor predicted worse RFS or survival within this subgroup (Additional file 1: Table S4).

### Association of high *IL2RA* expression with known prognostic mRNA expression biomarkers in intermediate-risk AML by NanoString

**Fig. 3:**
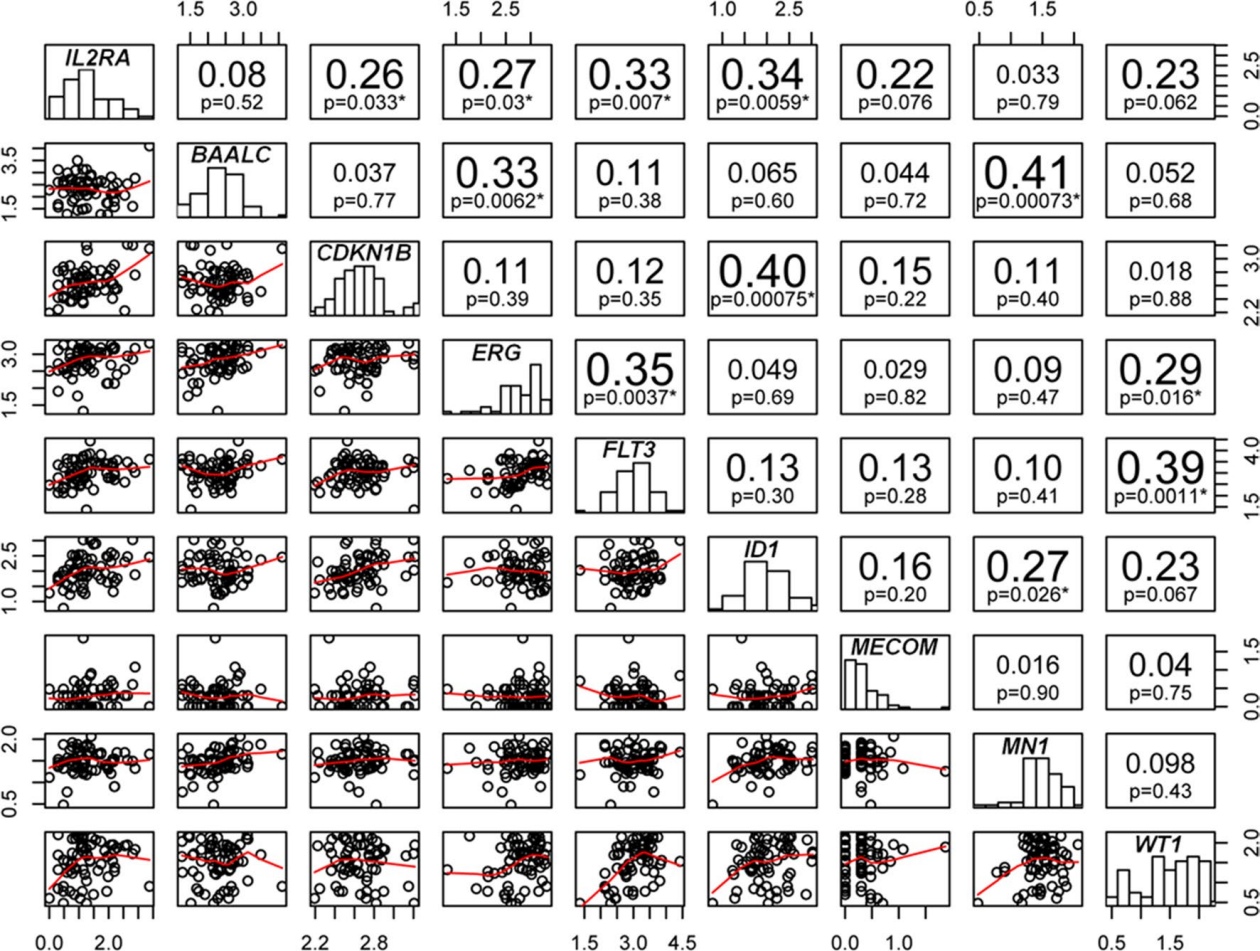
Correlation analysis of nine gene expression prognostic markers within intermediate-risk AML. The mRNA expressions of the nine AML gene expression prognostic genes were quantified by NanoString technology in 66 intermediate-risk AML. The correlations between the genes were done by bivariate correlation analysis. The scatter plots, the Spearman r and p values of the correlations are displayed. Correlations with p ≤ 0.05 were labeled by *

The prognostic value of many mRNA expression biomarkers was previously indicated in intermediate-risk AML and we sought to examine if *IL2RA* mRNA expression is correlated to these prognostic mRNA expression markers and if its prognostic value is independent of these biomarkers. We developed a NanoString multiplexed gene panel (Additional file 1: Table S5) and simultaneously measured the mRNA expressions of 8 prognostic genes (*BAALC*, *CDKN1B*, *ERG*, *MECOM/EVI1*, *FLT3*, *ID1*, *MN1* and *WT1)* as well as *IL2RA* in an intermediate-risk AML cohort 66 patients. The clinical characteristics of these patients were described in Additional file 1: Table S6. The mRNA expression of *IL2RA* were found to significantly associate with expression of four other prognostic genes including *ID1* (p = 0.006, Spearman R = 0.335, *FLT3* (p = 0.007, Spearman R = 0.329), *ERG* (p = 0.030, Spearman R = 0.267) and *CDKN1B* (p = 0.033, Spearman R = 0.263) (Fig. 3) in patients with intermediate-risk AML.

### Added prognostic value of high *IL2RA* mRNA expression to known mRNA expression prognostic biomarkers in independent intermediate-risk AML cohorts

In our intermediate-risk AML cohort based on NanoString assay, we further examined the prognostic value of *IL2RA* in the context of eight other mRNA expression biomarkers. The cutoff value for high vs. low expression of *IL2RA* was set at 80th percentile, for *MECOM/EVI1* at 90th percentile [46] and for the rest of genes at 50th percentile [47]. Univariable analyses showed that high *IL2RA, ERG, FLT3* and *WT1* mRNA expression levels correlated with worse clinical outcomes including RFS and OS in intermediate-risk AML (Table 7). In particular, high *IL2RA* mRNA level predicted shorter RFS (p < 0.001 and Table 7) and shorter OS (p = 0.004, Table 7) in this cohort of intermediate-risk AML. Further multivariable analysis showed that high *IL2RA* (p < 0.001, HR = 8.171) and *FLT3* (p = 0.008, HR = 3.314) mRNA expressions remained significant as predictors for shorter RFS, whereas high *ERG* (p = 0.008, HR = 5.541) and *IL2RA* (p = 0.044, HR = 2.765) mRNA expressions remained significant for shorter OS (Table 7). We also generated receiver operating characteristic (ROC) curves on the nine prognostic mRNA gene expression markers to test their performance in predicting relapse (Additional file 1: Figure S3) or survival (Additional file 1: Figure S4). The ROC curves showed superior performance of *IL2RA* in predicting relapse and survival than most other potential prognostic markers. In addition, the added prognostic value of *IL2RA* and *FLT3* was observed in predicting relapse (Additional file 1: Figure S3); *IL2RA* and *ERG* in predicting survival (Additional file 1: Figure S4).

**Table 7:** Uni- and multivariable analyses of multiple prognostic gene expression biomarkers in cytogenetically intermediate-risk AML

Prognostic markers	Univariable		Multivariable			
	RFS	OS	RFS		OS	
	p value	p value	p value	HR (95% CI)	p value	HR (95% CI)
*BAALC* high vs. low	0.070	0.927				
*CDKN1B* high vs. low	0.229	0.583				
*ERG* high vs. low	0.009	0.001			0.008	5.541 (1.566–19.602)
*MECOM/EVI1* high vs. low	0.513	0.409				
*FLT3* high vs. low	0.001	0.006	0.008	3.314 (1.372–8.008)		
*ID1* high vs. low	0.308	0.216				
*IL2RA* high vs. low	< 0.001	0.004	< 0.001	8.171 (2.750–24.279)	0.044	2.765 (1.028–7.437)
*MN1* high vs. low	0.847	0.211				
*WT1* high vs. low	0.002	0.006				

In the intermediate-risk AML subgroup (n = 80) of TCGA-LAML cohort, we also examined the prognosis value of the nine prognostic genes by uni- and multivariable analyses. In this independent study, high *IL2RA* mRNA expression level alone was shown to independently predict adverse outcomes including shorter EFS (p < 0.001 and HR = 3.276; Additional file 1: Table S7) and shorter OS (p < 0.001 and HR = 3.515; Fig. 2d and Additional file 1: Table S7). ROC curves on the nine mRNA gene expression markers were additionally generated and the ROC curve of *IL2RA* showed superior performance than other potential prognostic biomarkers in predicting clinical outcomes in the intermediate-risk AML subgroup within the TCGA-LAML cohort (Additional file 1: Figures S5, S6).

## Discussion

The prognostic value of CD biomarkers has been increasingly recognized in the AML research community over the last decade to help improve the current standard prognostic tools in AML clinical practice [2, 5, 14]. These CD markers usually predict inferior clinical outcome in AML. Specifically, some of them were shown to offer additional adverse prognostic value to current stratification strategy, such as CD25 and CD56 in cytogenetically intermediate-risk AML [5, 48] and CD56 in AML with t(8;21);*AML1/ETO* or with t(15;17);*PML/RARA* [14, 49]. While the majority of these CD biomarkers were investigated at protein level using flow cytometry technology, the prognostic value of these CD biomarkers at mRNA level remains largely unknown. Our study is a first study that emphasized on prognostic value of mRNA expressions of CD marker genes in a systemic manner and we established that mRNA expression of *IL2RA* gene, among a selected list of CD marker genes, is a significant and independent prognostic biomarker in AML, in particular, in CBF and intermediate-risk AML subtypes.

Our study quantified *IL2RA* mRNA expression in 239 AML excluding APL and showed that *IL2RA* mRNA is differentially expressed in groups classified by *FLT3*-*ITD*, *CEBPA*^*DM*^ or *NPM1*+*FLT3*-*ITD*− status, the recurrent genetic mutations that are prognostic relevant. Through a minimal p-value approach, we determined the optimal cutoff value for *IL2RA* mRNA expression that facilitate its use as a prognostic tool. We found that high *IL2RA* mRNA level is positively or negatively correlated with recurrent mutational and cytogenetic aberrations including *FLT3*-*ITD*, *NPM1*+*FLT3*-*ITD*−, and t(16;21);*FUS/ERG*, which was consistent with previous reports on CD25 [3, 5]. Our results by uni- and multivariable analyses clearly showed that high *IL2RA* mRNA expression was correlated with lower CR rate, shorter RFS and OS in AML and the effect is independent on other prognostic factors such as age, cytogenetic, *FLT3*-*ITD* and *c*-*KIT D816V* status. Therefore, we established the significant and independent role of high *IL2RA* mRNA expression as an adverse prognostic factor in AML.

While *IL2RA* could provided additional prognostic information to cytogenetics in AML, we further studied its prognostic value in AML of distinct cytogenetics risk statuses. CBF AML is a group of AML defined by carrying transcripts t(8;21);*AML1/ETO *or inv(16);*CBF/MYH11*. Although CBF AML belongs to the favorable-risk group, it still demonstrated considerable clinical, pathophysiological and molecular heterogeneity. In CBF AML, around 85% cases achieve CR after induction therapy, about 40–50% cases relapse after CR and around 50% cases remain alive at 5 years [50, 51]. In previous studies, the prognostic value of CD25 protein expression in favorable-risk AML or CBF AML has not been reported probably due to the low frequency of CD25 positive cases in favorable-risk group in the cohorts [5, 7]. Although the frequency of high expression *IL2RA* cases was also low in APL (2 out of 63, Additional file 1: Table S8) and in all favourable-risk cases in our study, the frequency of high *IL2RA* mRNA expression cases in CBF AML (16.7%) was not significantly different from that in intermediate-risk and poor-risk AML. Further survival analyses within this subgroup of patients demonstrated strong association between high *IL2RA* expression and shorter RFS and OS, and it is the first time that elevated expression of CD25 biomarker gene are indicated in predicting adverse outcome in CBF AML.

Mutations in genes activating tyrosine kinase signaling (including *c*-*KIT*, *N/KRAS*, and *FLT3*) have been shown as the most frequent additional mutations in CBF AML that confer worse prognosis [51]. Of these mutations, the *c*-*KIT D816V* has been indicated to predict poor prognosis, particularly in AML with t(8;21) [51, 52]. Correlation analysis in our study in CBF AML showed no evidence of correlation between high *IL2RA* expression and *c*-*KIT D816V* status (p = 1.000). Further uni- and multivariable survival analysis indicateded that high *IL2RA* expression and *c*-*KIT D816V* mutation remained as two independent prognostic factors to predict shorter RFS, whereas high *IL2RA* expression alone remained significant in predicting shorter OS in CBF AML. Moreover, high *IL2RA* expression significantly correlated with *FLT3*-*ITD* mutation in CBF AML (p = 0.023), similar as in intermediate-risk AML. However, it should be noted that since *FLT3*-*ITD* mutation was present at a much lower frequency in CBF-AML (4.7% in our study and 7% in literature) than in intermediate-risk AML [51], the interaction between high *IL2RA* expression and *FLT3*-*ITD* mutation should be rather limited. It remained to be studied if the prognostic significance of high *IL2RA* mRNA expression in CBF AML is associated with gene mutations involved in activating tyrosine kinase signaling other than *FLT3*-*ITD* or *c*-*KIT D816V mutation,* or with other mechanisms. Nevertheless, our results suggested the significant potential of high *IL2RA* mRNA, in coordination with *c*-*KIT D816V mutation,* to further refine prognostification in CBF AML and warrant future validation in larger cohort of CBF-AML.

The prognostic value of *IL2RA* or CD25 in intermediate-AML has been consistently shown in our study and previous studies [5–7]. However, it was unknown if *IL2RA* prognostic value is associated with or dependent on other gene expression prognostic biomarkers which has been well studied in intermediate-risk or normal karyotype AML. In our intermediate-risk AML cohort, a panel of prognostic gene expression markers including *BAALC* [15], *CDKN1B* [15], *ERG* [15, 18], *FLT3* [53], *ID1* [17, 47], *IL2RA, MN1* [15, 19], *MECOM/EVI1* [46], and *WT1* [54, 55] were quantified by NanoString technology. Despite the strong correlations of *IL2RA* expression with other mRNA biomarkers were shown, *IL2RA* and *FLT3* mRNA expressions remained significant in predicting shorter RFS, whereas *ERG* and *IL2RA* mRNA expressions remained significant in predicting shorter OS by uni- and multivariable analyses. Independent analysis on the same panel of genes in intermediate-risk AML cases in TCGA-LAML cohort consistently demonstrated the significant and independent value of *IL2RA* mRNA expression in predicting inferior clinic outcome. So far, various mRNA expression gene-panels have been designed for better stratification in AML [22, 47] and our results support the incorporation of *IL2RA* gene into such multi-gene panel to improve prognostification within intermediate-risk AML.

The mechanisms by which *IL2RA* is prognostic in AML can be implicated by its correlation results with other mRNA expression biomarkers. It is first shown by our study that upregulation of *IL2RA* was correlated with upregulation of *FLT3* tyrosine kinase transcripts [53] and of transcription factor *ID1*, a key common target of oncogenic tyrosine kinases that contribute to transformation of leukemias [56], indicating a strong association of *IL2RA* expression with tyrosine kinases pathways. Our study also showed significant correlation of *IL2RA* with *ERG* and *CDKN1B*, the expressions of which have been indicated in stem cell-like featured gene signatures [57, 58]. Previously, it has been proposed that CD25 is a surrogate marker for leukemia stem cell (LSC) [4, 5], our results again provided evidence that *IL2RA/CD25 *is involved in the crosstalk of LSC related signalling and support that *IL2RA* is an indicator of the LSC signature which has been shown as a fundamental adverse prognostic feature in AML [59]. In addition to these cell autonomous mechanisms, there may be non-cell autonomous mechanisms that contribute to the prognostic role of *IL2RA* mRNA expression such as *IL2RA* expression regulatory T cells [60] or IL2/IL3 interplay [61], which warrant future studies.

## Conclusions

In conclusion, our findings established the significant and independent prognostic value of high *IL2RA* mRNA expression in AML, particularly in CBF and intermediate-risk AML, supporting the application of high *IL2RA* mRNA expression as a prognostic tool to improve current stratification strategies in AML.

## Additional file

**Additional file 1.** Additional tables and figures.

## Data Availability

The datasets used and/or analysed during the current study are available from the corresponding author on reasonable request.

## Abbreviations

AML: acute myeloid leukemia
APL: acute promyelocytic leukemia
BM: bone marrow
CBF: core binding factor
CD: cluster of differentiation
CR: complete remission
*CXCR4*: *C*-*X*-*C chemokine receptor type 4*
Fab: French–American–British
FCM: flow cytometry
*NPM1*: *nucleophosmin 1*
*FLT3*-*ITD*: FMS-like tyrosine kinase 3-internal tandem duplication
HCT: hematopoietic stem cell transplantation
HR: hazard ratio
*IL2RA*: *interleukin 2 receptor subunit alpha*
RQ-PCR: realtime quantitative-polymerase chain reaction
RFS: relapse-free survival
OS: overall survival
