# Supplementary material for: High IL2RA mRNA expression is an independent adverse prognostic biomarker in core binding factor and intermediate-risk acute myeloid leukemia
Source: J Transl Med. 2019 Jun 6;17:191. doi: 10.1186/s12967-019-1926-z (PMC6551869; doi:10.1186/s12967-019-1926-z)
Supplement: Supplementary file 1 — Additional file 1. Additional tables and figures. [file 12967_2019_1926_MOESM1_ESM.docx]

**Figure S1. Correlation between *IL2RA* mRNA expression and BM blast percentage**

**
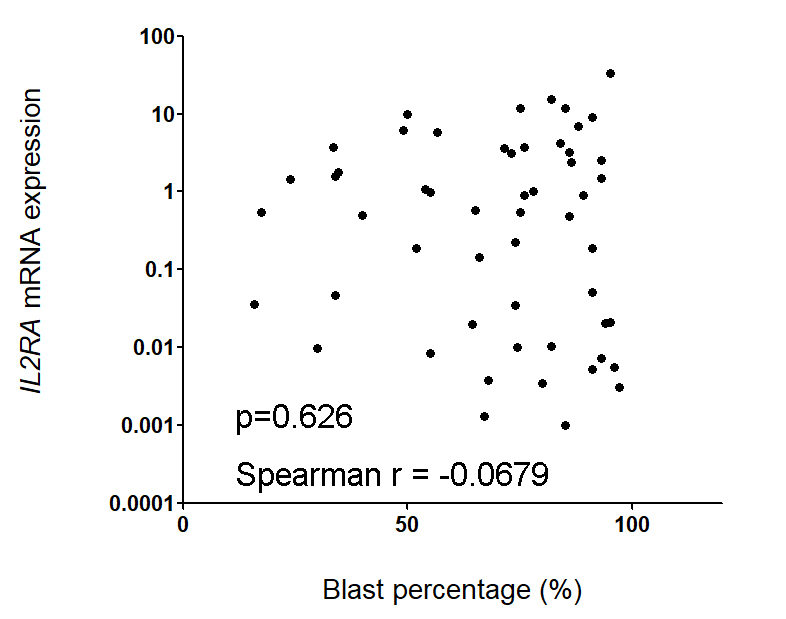
**

**Figure S2. Correlation between CXCR4 protein expression by FCM and mRNA expression by RQ-PCR**

**
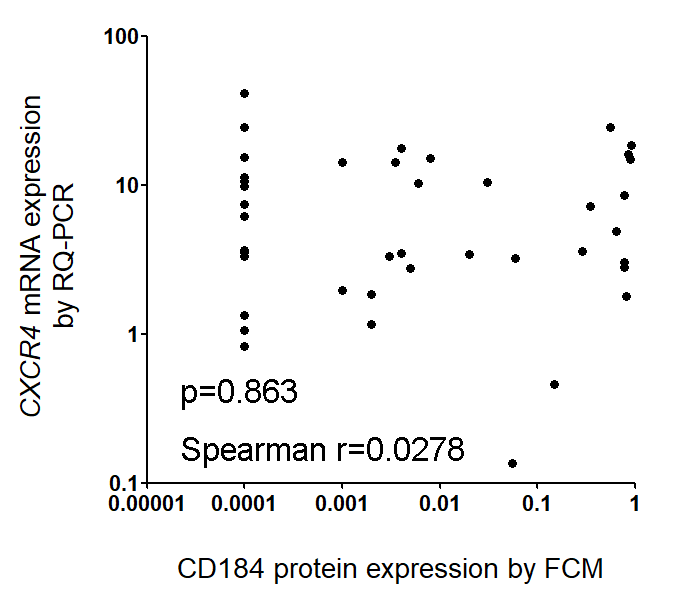
**

**Figure S3. ROC curves of gene expression markers in predicting relapse in intermedaite-risk AML (n=66)**

**
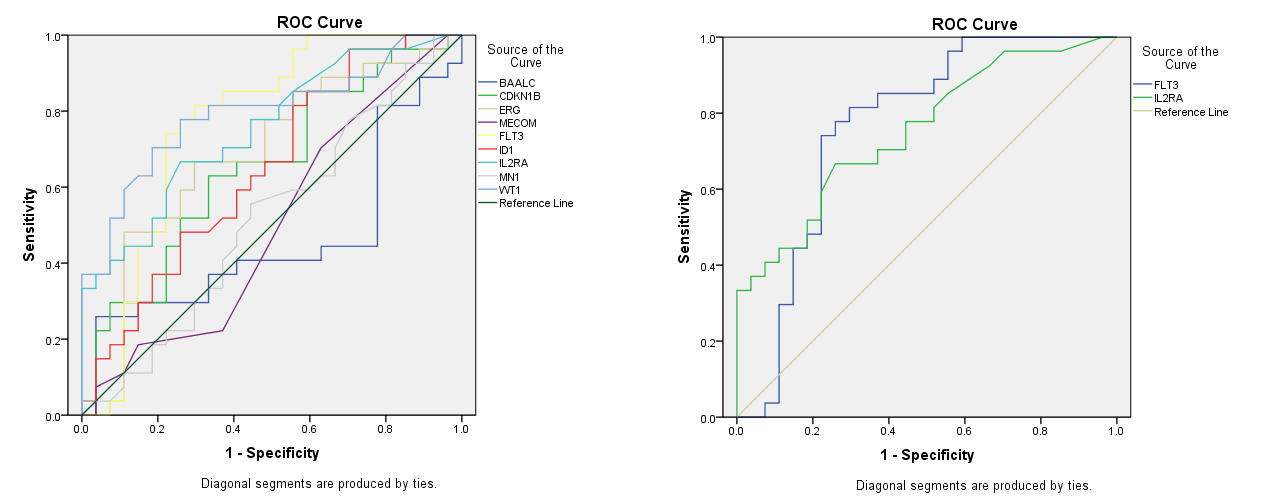
**

The AUC of *IL2RA* ROC curve is 0.754 and the AUC of *FLT3* ROC curve is 0.765.

**Figure S4. ROC curves of mRNA gene expression biomarkers in predicting survival in intermedaite-risk AML (n=66)**

**
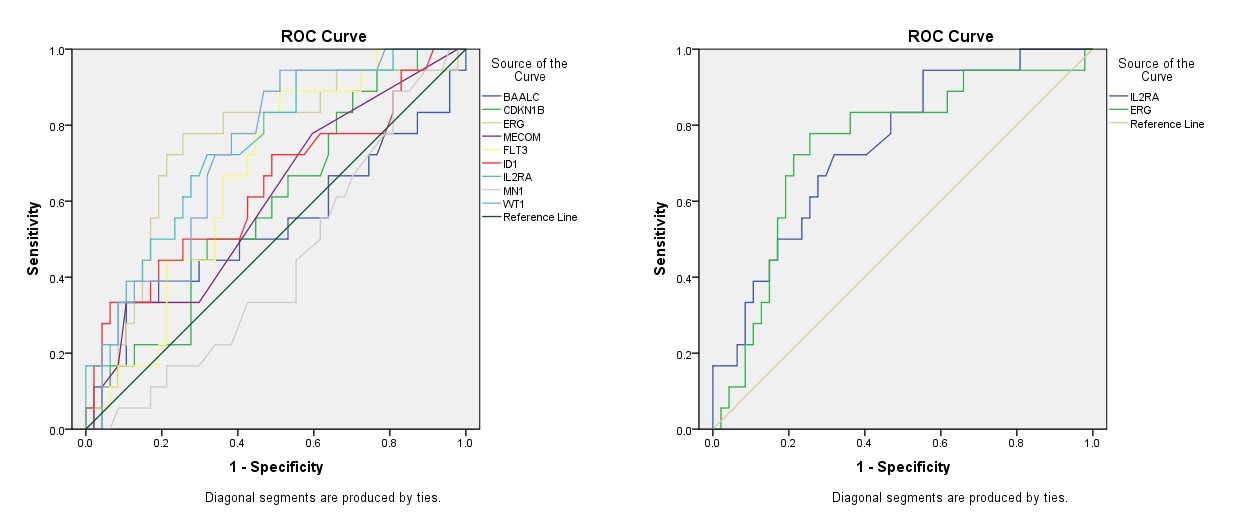
**

The AUC of *IL2RA* ROC curve is 0.747 and the AUC of *ERG* ROC curve is 0.746.

**Figure S5. ROC curves of mRNA gene expression biomarkers in predicting events in TCGA-LAML intermedaite-risk AML (n=80)**

**
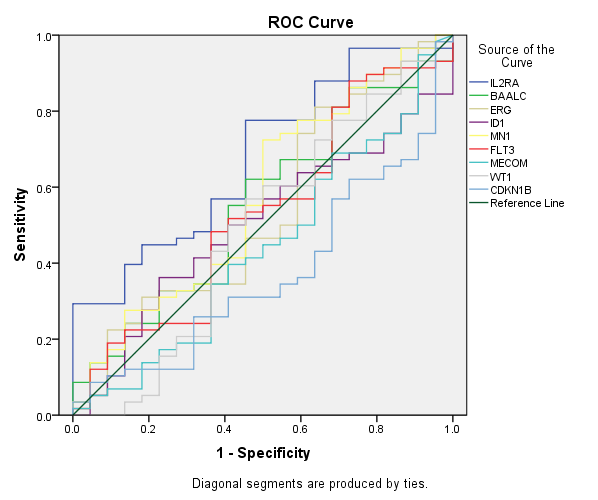
**

The AUC of *IL2RA* ROC curve is 0.776.

**Figure S6. ROC curves of gene expression markers in predicting survival in TCGA-LAML intermedaite-risk AML**

**
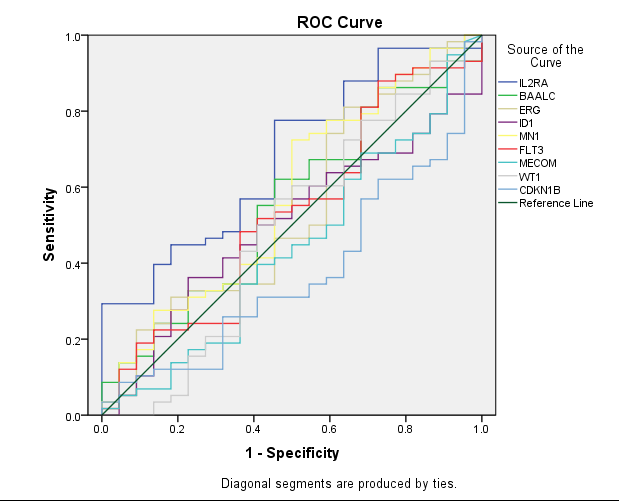
**

The AUC of *IL2RA* ROC curve is 0.678.

**Table S1. Evaluation of the prognostic potential of** **mRNA expressions of four prognostic CD markers genes**

|  | Correlation of mRNA/protein expressions | | Univariable analysis on OS in TCGA-LAML cohort | | |
| --- | --- | --- | --- | --- | --- |
|  | Our studies | Reported studies | AML  (n=151) | Non-APL AML (n=136) | Intermediate AML (n=80) |
| *IL2RA/CD25* | p <0.001;  Spearman r=0.893 (n=54) |  | p<0.001 | p= 0.001 | p<0.001 |
| *CXCR4/CD184* | p=0.863;  Spearman r=0.0278 (n=41) |  | p= 0.187 | p= 0.247 | p= 0.905 |
| *CD34* |  | Ref 1: p<0.001;  Spearman r=0.545 (n=72)  Ref 2: p<0.01;  Pearson r=0.82 (n=73) | p= 0.052 | p= 0.331 | p= 0.182 |
| *CD56* |  | Ref 1: p=0.180;  Spearman r=0.126 (n=73)  Ref 2: p<0.01;  Pearson r =0.80 (n=73) | p= 0.471 | p= 0.862 | p= 0.283 |

Ref 1: reference 1 by [Kern *et al.*](#_ENREF_2)[^40^](#_ENREF_40) Ref 2: reference 2 by [Fernandez *et al.*](#_ENREF_1) [^39^](#_ENREF_39)^,^

**Table S2. Analsyis on clinical outcome to determine optimal *IL2RA* expression cutoff value**

| Cutoff percentile | P-Value | | | |
| --- | --- | --- | --- | --- |
|  | CR status | Relapse status | RFS | OS |
| 90% | 0.099 | 0.011 | <0.001 | 0.003 |
| 85% | 0.100 | 0.002 | <0.001 | <0.001 |
| 80% | 0.005 | 0.001 | <0.001 | <0.001 |
| 75% | 0.005 | 0.002 | <0.001 | 0.001 |
| 70% | 0.048 | 0.003 | <0.001 | 0.014 |

The P-value for CR and Relapse status was calculated by logistic regression, the P-value for RFS and OS were calculated by Kaplan-Merier survival analysis

**Table S3. Correlation of *IL2RA* mRNA level with patients’ characteristics** **in AML cohort**

| **e** | G N | Percent  age | High *IL2RA* mRNA level | Low *IL2RA* mRNA level | P-value |
| --- | --- | --- | --- | --- | --- |
| Total | 239 |  |  |  |  |
| Age Group |  |  |  |  | 0.447 |
| 15~29 | 65 | 27.2% | 14 | 51 |  |
| 30~49 | 113 | 47.3% | 19 | 94 |  |
| 50~65 | 61 | 25.5% | 15 | 46 |  |
| Gender |  |  |  |  | 1.000 |
| Male | 127 | 53.1% | 26 | 101 |  |
| Female | 112 | 46.9% | 22 | 90 |  |
| Blast Percentage |  |  |  |  | 0.243 |
| ≥50% | 149 | 62.3% | 26 | 124 |  |
| 20~49% | 90 | 37.7% | 22 | 68 |  |
| WBC count |  |  |  |  | 1.000 |
| ≥30 billion/L; high | 58 | 34.7% | 12 | 46 |  |
| <30 billion/L; low | 109 | 65.3% | 23 | 86 |  |
| AML Type  De novo AML  Secondary AML | 231  8 | 96.7% 3.3% | 45  3 | 187  5 | 0.202 |
| Fab Subtypes |  |  |  |  |  |
| Fab M0 | 6 | 2.6% | 0 | 6 | 0.092 |
| Fab M1 | 32 | 13.6% | 6 | 26 |  |
| Fab M2 | 118 | 50.2% | 18 | 100 |  |
| Fab M4 | 43 | 18.3% | 10 | 33 |  |
| Fab M5 | 26 | 11.1% | 6 | 20 |  |
| Fab M6 | 10 | 4.3% | 5 | 5 |  |
| ND | 4 |  |  |  |  |
| Cytogenetics groups |  |  |  |  | 0.667 |
| Favorable-risk (CBF) | 54 | 22.6% | 9 | 45 |  |
| Intermediate-risk | 148 | 61.9% | 30 | 118 |  |
| Poor-risk | 37 | 15.5% | 9 | 28 |  |

ND: not determined.

**Table S4. Univariable analysis on RFS and OS in poor-risk AML**

| Variable |  | RFS | OS |
| --- | --- | --- | --- |
|  |  | P value | P value |
| Age group |  | 0.512 | 0.447 |
| High WBC counts |  | 0.527 | 0.550 |
| High Blast Percentage |  | 0.849 | 0.599 |
| Secondary AML |  | 0.531 | 0.797 |
| High *IL2RA* mRNA level |  | 0.868 | 0.666 |
| *FLT3-ITD+* |  | 0.332 | 0.525 |

**Table S5 NanoString probes designed for AML prognostic gene expression assay**

| Gene symbol | Accession | NanoString probe sequence | Tm_CP | Tm_RP |
| --- | --- | --- | --- | --- |
| *BAALC* | NM_024812.2 | AAGAATCACAAAGAACTGTGTCAACTAGCAGAGAGTCCAAGCAGAAGGGCAGATGGACTTCTTCAGTGTCCTTCACGGCACTGGATCCCATCAAAGAACC | 80 | 81 |
| *CDKN1B* | NM_004064.2 | GCTTCCGAGAGGGGTTCGGGCCGCGTAGGGGCGCTTTGTTTTGTTCGGTTTTGTTTTTTTGAGAGTGCGAGAGAGGCGGTCGTGCAGACCCGGGAGAAAG | 82 | 81 |
| *ERG* | NM_182918.3 | CTCCACTACCTCAGAGAGACTCCTCTTCCACATTTGACTTCAGATGATGTTGATAAAGCCTTACAAAACTCTCCACGGTTAATGCATGCTAGAAACACAG | 81 | 82 |
| *EVI1* | NM_005241.2 | CTGCGGCGGAATCCAGTGCTATCCAGTCCATAAGCCACGTATGACGTTATCAAGGTTGACCAGAGTGGGACCAAGTCCAACAGTAGCATGGCTCTTTCAT | 81 | 82 |
| *FLT3* | NM_004119.1 | AAATGGAAAACCAGGACGCCCTGGTCTGCATATCTGAGAGCGTTCCAGAGCCGATCGTGGAATGGGTGCTTTGCGATTCACAGGGGGAAAGCTGTAAAGA | 82 | 80 |
| *ID1* | NM_002165.2 | CTGCCCCAGAACCGCAAGGTGAGCAAGGTGGAGATTCTCCAGCACGTCATCGACTACATCAGGGACCTTCAGTTGGAGCTGAACTCGGAATCCGAAGTTG | 82 | 81 |
| *IL2RA* | NM_000417.1 | CTTGGTAAGAAGCCGGGAACAGACAACAGAAGTCATGAAGCCCAAGTGAAATCAAAGGTGCTAAATGGTCGCCCAGGAGACATCCGTTGTGCTTGCCTGC | 79 | 82 |
| *MN1* | NM_002430.2 | CACGGAGGGTGACGAACCAAGGAGCCGTCGACTCGCTGGAATACAATTACCCGGGCGAGGCGCCCTCGGGACATTTTGACATGTTTTCGCCCTCTGACTC | 82 | 82 |
| *WT1* | NM_000378.3 | TCAGAGAGCAAGGCATCGGGGGTGAATCTTGTCTAACATTCCCGAGGTCAGCCAGGCTGCTAACCTGGAAAGCAGGATGTAGTTCTGCCAGGCAACTTTT | 82 | 83 |
| *ABL1* | NM_005157.3 | CTGCGTGAGCTATGTGGATTCCATCCAGCAAATGAGGAACAAGTTTGCCTTCCGAGAGGCCATCAACAAACTGGAGAATAATCTCCGGGAGCTTCAGATC | 78 | 81 |

Tm, Temperature; CP, capture probes; RP, Reporter probes

**Table S6.** **Clinical characteristics of our intermediate-risk AML cohort**

| Intermediate-risk patients | No. (%) |
| --- | --- |
| Total | 66 (100) |
| Age  15~29  30~49  50~70 | 12 (18.2)  33 (50.0)  21 (31.8) |
| Sex  Male  Female | 36 (54.5)  30 (44.5) |
| FAB subtype  M0  M1  M2  M4  M5  M6 | 2 (3.0)  15 (22.7)  28 (42.4)  9 (13.6)  9 (13.6)  3 (4.5) |
| Cytogenetic subgroup  Normal Karyotype  Trisomy 8  Trisomy Y  t(2;14)  t(9;11)  t(7;8)  t(16;21)  inv(7)  del(20)  del(12) | 53 (80.3)  3 (4.5)  2 (3.0)  2 (3.0)  1 (1.5)  1 (1.5)  1 (1.5)  1 (1.5)  1 (1.5)  1 (1.5) |

**Table S7. Uni- and multivariable analysis on EFS and OS in TCGA-LAML intermediate-risk AML**

|  | Univariable | |  | Multivariable | |  | |
| --- | --- | --- | --- | --- | --- | --- | --- |
| Variable | EFS | OS |  | EFS | | OS | |
|  | P value | P value |  | P value | HR(95%CI) | P value | HR(95%CI) |
| *BAALC* High vs. Low | 0.242 | 0.268 |  |  |  |  |  |
| *CDKN1B* High vs. Low | 0.690 | 0.497 |  |  |  |  |  |
| *ERG* High vs. Low | 0.249 | 0.863 |  |  |  |  |  |
| *MECOM/EVI1* High vs. Low | 0.367 | 0.483 |  |  |  |  |  |
| *FLT3* High vs. Low | 0.553 | 0.689 |  |  |  |  |  |
| *ID1* High vs. Low | 0.164 | 0.100 |  |  |  |  |  |
| *IL2RA* High vs. Low | <0.001 | <0.001 |  | <0.001 | 3.276 (1.759~6.100) | <0.001 | 3.515 (1.898~6.509) |
| *MN1* High vs. Low | 0.706 | 0.833 |  |  |  |  |  |
| *WT1* High vs. Low | 0.218 | 0.737 |  |  |  |  |  |

**Table S8. Distribution of high or low *IL2RA* expression level cases in APL and non APL patients**

|  | Total | High *IL2RA* mRNA | Low *IL2RA* mRNA | Fisher’s Exact P-value |
| --- | --- | --- | --- | --- |
| APL | 63 | 2 | 61 | <0.001 |
| Non APL | 239 | 48 | 191 |  |
| Total |  | 50 | 252 |  |
